# Bacteriocinogenic probiotic bacteria isolated from an aquatic environment inhibit the growth of food and fish pathogens

**DOI:** 10.1038/s41598-022-09263-0

**Published:** 2022-04-01

**Authors:** Wellison Amorim Pereira, Anna Carolina M. Piazentin, Rodrigo Cardoso de Oliveira, Carlos Miguel N. Mendonça, Yara Aiko Tabata, Maria Anita Mendes, Ricardo Ambrósio Fock, Edson Naoto Makiyama, Benedito Corrêa, Marisol Vallejo, Elias Figueroa Villalobos, Ricardo Pinheiro de S. Oliveira

**Affiliations:** 1 Laboratory of Microbial Biomolecules, School of Pharmaceutical Sciences, University of São Paulo, Rua Do Lago, 250, Cidade Universitária, São Paulo 05508-000 Brazil; 2 Fishing Institute of São Paulo/Salmoniculture Experimental Station, Av. Campos Do Jordão, Residencial Horto Florestal, Campos do Jordão, São Paulo 12460-000 Brazil; 3 Chemical Engineering Department, University of São Paulo, Rua Do Lago, 250, Cidade Universitária, São Paulo 05508-000 Brazil; 4 Laboratory of Experimental Hematology, University of São Paulo, Av. Prof. Lineu Prestes, 580, Cidade Universitária, São Paulo 05508- 000 Brazil; 5 Laboratory of Toxigenic Fungi and Mycotoxins, Av. Prof. Lineu Prestes, 1.374, Edifício Biomédicas II, 05508-900 São Paulo, Brasil; 6 Bacterial Biotechnology Laboratory, Faculty of Natural Sciences and Health Sciences, UNPSJB, Sede Trelew, Chubut, Argentina; 7 Nucleus of Research in Food Production, Faculty of Natural Resources, Catholic University of Temuco, Temuco, Chile

**Keywords:** Biotechnology, Microbiology, Molecular biology

## Abstract

The conditions of aquatic environments have a great influence on the microbiota of several animals, many of which are a potential source of microorganisms of biotechnological interest. In this study, bacterial strains isolated from aquatic environments were bioprospected to determine their probiotic profile and antimicrobial effect against fish and food pathogens. Two isolates, identified via 16S rRNA sequencing as *Lactococcus lactis* (L1 and L2) and one as *Enterococcus faecium* 135 (EF), produced a bacteriocin-like antimicrobial substance (BLIS), active against *Listeria monocytogenes, Salmonella* Choleraesuis and *Salmonella* Typhimurium. Antimicrobial activity of BLIS was reduced when exposed to high temperatures and proteolytic enzymes (trypsin, pepsin, papain and pancreatin). All strains were sensitive to 7 types of antibiotics (vancomycin, clindamycin, streptomycin, gentamicin, chloramphenicol, rifampicin and ampicillin), exhibited a high rate of adherence to Caco-2 cells and expressed no hemolysin and gelatinase virulence factors. EF showed some resistance at pH 2.5 and 3.0, and L2/EF showed higher resistance to the action of bile salts. Finally, the presence of bacteriocin genes encoding for proteins, including Nisin (L1 and L2), Enterocin A, B, P, and Mundticin KS (EF) was detected. The molecular and physiological evidence suggests that the bacterial isolates in this study could be used as natural antimicrobial agents and may be considered safe for probiotic application.

## Introduction

Probiotics are defined as live microorganisms, which when administered in adequate amounts confer a health benefit on the host^1^. However, to be considered a probiotic, these microorganisms must undergo experiments to attest safety for use in food. Probiotics isolated from aquatic animals are spread through water and via other living organisms, and once they reach the host's intestines, these microorganisms perform vital functions. Several anatomical structures are potential growth sites for microorganisms, such as the skin, gills and gastrointestinal tract^2,3^. Intestinal content is thus an important source of potential probiotic microorganisms that can subsequently be used as food supplements^4^.

Proper nutrition is intrinsically associated with correct development and efficient immunological defenses. Thus, studies have shown that both in humans and animals, the microbiota plays an essential role in the proper development and defense against pathogens^2^. Probiotic use in feed improves the health of aquatic animals, without the presence of negative side-effects^5^. Among the studies that have demonstrated the benefits of probiotic use, different mechanisms of action have been noted, that differ according to the species and environmental conditions that the microorganism encounters^6,7^. Probiotics used in aquaculture have included specific strains of yeasts and especially bacteria, including representatives of *Lactococcus* sp., *Enterococcus* sp., among others^8^. Some species belonging to the lactic acid bacteria (LAB) are considered safe (GRAS, Generally Reported as Safe)^9^ and can be producers of natural antimicrobials, such as bacteriocins^7^.

LAB are commonly recommended for aquaculture, and dietary supplementation results in an improved activity of digestive enzymes, immune response, development and even water quality^4,10^. Stimulation of the production of digestive enzymes, such as amylase, protease, lipase and lysozyme, can be an important consequence of probiotic use^11^. In healthy animals, these enzymes are intrinsically associated with improved digestibility, nutritional intake and weight gain^12,13^. Colonization induction and the development of beneficial strains in the intestinal tract also lead to the production of other beneficial substances in addition to enzymes^2^.

As previously mentioned, an important characteristic of LAB is the ability to produce bacteriocins that play a key role in controlling pathogens^14^. These are conceptualized as small, cationic, heterogeneous, hydrophobic antimicrobial peptides produced by different microorganisms, with high isoelectric points, an amphipathic character, and a variety of modes of action and biochemical properties^14,15^. Since 1925, with the discovery of colicin, research on bacteriocins has received great attention^16^ and by 1995 more than a hundred different types of bacteriocins had been identified^17^. Bacteriocins provide an important competitive advantage for the species that produce them^18^. Probiotics of interest can remain in the intestinal tract while producing bacteriocins, exerting synergistic effects, since they are not toxic to the host and the LAB exert various beneficial functions^19^. Most of the bacteriocins that have been tested to date were isolated from LAB and are generally used in foods for their high antimicrobial potential^18^.

The major goal of producing bacteriocins is to increase bacteria's competition for food and ecological niches in the microbiota ^18^. The antimicrobial effect of bacteriocins is related to their action on anionic lipids present in the membrane, which results in the formation of pores as well as disrupting ATP synthesis and amino acid transport^20^. For this reason, most studies evaluating bacteriocins are carried out with Gram-positive bacteria, as they have membranes that are richer in anionic lipids. The same effect can be observed in Gram-negatives; however, bacteriocin needs to cross the complex structure of the outer membrane^18^. An example is the bacteriocin microcin C7–C51 which has already been described to be effective against strains of the genus *Escherichia*, *Enterobacteria*, *Klebsiella*, *Salmonella*, *Shigella*, *Proteus*, among others^14^. Studies also point to the possibility of using bacteriocins as an alternative to combat antibiotic-resistant microorganisms, since their mode of action is different^20^.

Bacterial diseases can affect various sectors, such as food production and fish farming. In this regard, some pathogens of interest belong to the genus *Streptococcus*, *Staphylococcus*, *Listeria* and *Salmonella*. Streptococcosis is a disease caused by the genus *Streptococcus* and it is triggered by stress and high density in fish culture, which can lead to considerable production losses^21^. Staphylococcal outbreaks are food poisoning caused by *Staphylococcus aureus*, an enterotoxins producing bacteria. Despite not being part of the microbiota of aquatic animals, its presence may be associated with diseases^22^. Thus, bacteria of the *Salmonella* genus are important pathogens known in the literature for their dissemination via water and/or contaminated food and difficult control^23^. Finally, *Listeria monocytogenes* is a pathogen difficult to control with a high incidence in fish processing facilities and has shown some resistance to several antimicrobials^24^.

Experiments with aquatic animals have yielded promising results and feed supplementation effectiveness can be optimized if different approaches for the use of probiotics are tested^25^. Recent studies have shown that the future of probiotic research in aquaculture lies in the use of new supplementation techniques, such as the mixing of two or more strains. Indeed, mixing different probiotic microorganisms increases the product efficacy, which opens up the possibility of researching new lines aimed at investigating the interaction of these microorganisms as well as their joint action for the benefit of animal health. But as few examples have been analyzed in detail, specific studies are needed to test each of the strains used and their impact on individual animal models^2^.

Therefore, the aim of this study was to evaluate the probiotic and bacteriocinogenic potential of bacteria isolated from an aquatic environment and their antimicrobial potential against important fish and food pathogens.

## Materials and methods

### Sampling and ethical aspects

Samples were obtained by field collection carried out at the Salmoniculture Experimental Station of the São Paulo Fishing Institute (Campos do Jordão, Brazil). Rainbow trout (*Oncorhynchus mykiss*), approximately 16 weeks old, were selected for the start of bioprospecting. After capture, the animals were sacrificed respecting biosafety and anesthesia rules validated by the institutions themselves, and then, under aseptic conditions, the cecum was removed, stored in a sterile flask in thermal boxes (~ 4 °C), and transported to the laboratory for immediate analysis. This study was analyzed and approved by the Ethics Committee of São Paulo Fishing Institute (registration number 07/2020). For fish anesthesia, an aqueous solution of benzocaine (100 mg/L^−1^) was used until the loss of balance and reduction of opercular movements. Testing was done following guidelines and regulations.

EF was obtained from the collection belonging to the Laboratory of Bacterial Biotechnology (Universidad Nacional de la Patagonia, Argentina). The strain was isolated from starfish (order *Forcipulatida*) in Playa Unión, Rawson-Chubut (Patagonia, Argentina) and donated by Prof. Marisol Vallejo, National University of Patagonia San Juan Bosco (Argentina).

### Bioprospecting and identification by biochemical tests and MALDI-TOF

The protocols described below were used for the isolation and identification of samples present in the cecum content of rainbow trout and starfish. The isolation was carried out according to the methods described by Schirru et al.^26^ with minor modifications. Samples of 25 g of excrement were homogenized in 225 mL of peptone water in a Stomacher. Serial dilutions were performed and cultivated in Man, Rogosa and Sharpe (MRS) and M17 media (BD Difco, New Jersey, USA) with cycloheximide (0.1 g/L). The plates were incubated under different temperatures (15, 25, 32 and 37 °C), for up to 48 h in anaerobic and aerobic conditions. After this period, approximately 300 CFUs were randomly chosen on each plate and replicated in the same culture medium and conditions. Then, biochemical tests were carried out for the classification of isolated microorganisms, such as Gram test (Gram method), production of Catalase (addition of hydrogen peroxide), and analysis by MALDI-TOF (Optical Microscopy and Ionization Mass Spectrometry by Laser Desorption Matrix assisted with flight time analyzer). For MALDI-TOF analysis, isolates defined as Gram-positive, Catalase-negative and with morphology corresponding to cocci and/or bacilli were selected. The protocol described by Alves et al.^27^ was used for this test.

Therefore, the isolated strains were grown according to their isolation conditions in plates with 1.5% MRS/M17 medium for 24 h, as previously described. Isolates and 200 μL of sterile distilled water were mixed into a 1.5 mL microtube, being homogenized for 1 min using a vortex device. A volume of 900 μL of ethanol was transferred into the tubes, and centrifugated at 12,000×*g* for 5 min. The supernatant was discarded, and the samples were dried at room temperature for the loss of alcohol residues. 50 μL of formic acid (70%) and 50 μL of acetonitrile were added to the tubes, with a vortex homogenization. Subsequently, a matrix of α-cyano-4-hydroxycinnamic acid was prepared as a solution saturated in 50% acetonitrile and 2.5% trifluoroacetic acid. In a steel target plate, 1μL of treated samples and 1μL of matrix solution was added for drying at room temperature. Finally, the selected strains were cryopreserved in glycerol (20% v/v) at – 80 °C. For identification by mass spectrometry, the ItrafeXtreme MALDI-TOF equipment (Bruker Daltonics, Germany) was used, operating in the positive linear ion mode. The mass spectra were acquired in a mass range of 2 to 20 kDa with ions formed by intelligent beam radiation using a frequency of 2000 Hz, PIE 100 ns, 7 kV lens. The voltages for the first and second ion sources were 25 kV and 3 kV, respectively. The bacteria were identified using the Biotyper 3.1 database. Cut-off values greater than 2 and 1.7 were used to identify species and genera, respectively^27^.

### 16S rRNA sequencing

For the identification of species at the molecular level, isolates L1, L2 and EF were subjected to partial sequencing of the 16S gene (rRNA) using the following primers: (PLB16) AGAGTTTGATCCTGGCTCAG and (MLB16) GGCTGCTGGCACGTAGTTAG. Genomic DNA was extracted using the PrepMan Ultra® kit protocol (Applied Biosystems, Carlsbad, CA, USA), following the manufacturer’s instructions. The DNA was quantified using a NanoDrop 2000 spectrophotometer (Thermo Fisher Scientific, Wilmington, DE, USA) and used for amplification reactions with PCR Master Mix (Promega, San Luis Obispo, CA, USA) under the following thermal cycling conditions: 94 °C for 5 min, followed by 35 cycles at 94 °C for 1 min, 55 °C and 72 °C for 1 min, followed by a final extension of 7 min at 72 °C. PCR products were purified with a QIAquick PCR Purification kit (Qiagen, Hilden, Germany) and sequenced in both directions using a Big Dye® Terminator v3.1 Cycle Sequencing Kit (Applied Biosystems). After contig assembly and edition, 16S sequences were used to conduct BLAST search analysis for species identification. All sequences generated in this study were deposited in the GenBank database (Table 1).

**Table 1:** Molecular identification (16S rRNA) and screening for presence of bacteriocin genes in *L. lactis* (L1 and L2) and *E. faecium* strains (EF).

Strains	Molecular identification	Accession number	Bacteriocin genes	Results	Reference
L1	*Lactococcus lactis*	MZ926851	Nisin	+	Alegría et al. ^59^
			Lacticin 3147	−	Alegría et al. ^59^
			Lacticin 481	−	Alegría et al. ^59^
			Lactococcin 972	−	Martínez et al. ^60^
			Lactococcin A, B, M	−	Alegría et al. ^59^
			Lactococcin G and Q	−	Alegría et al. ^59^
L2	*Lactococcus lactis*	MZ926852	Nisin	+	Alegría et al. ^59^
			Lacticin 3147	−	Alegría et al. ^59^
			Lacticin 481	−	Alegría et al. ^59^
			Lactococcin 972	−	Martínez et al. ^60^
			Lactococcin A, B, M	−	Alegría et al. ^59^
			Lactococcin G and Q	−	Alegría et al. ^59^
EF	*Enterococcus faecium*	MZ735396	Enterocin A	+	De Vuyst ^61^
			Enterocin B	+	De Vuyst ^61^
			Enterocin P	+	De Vuyst ^61^
			Enterocin LB50A	−	De Vuyst ^61^
			Enterocin LB50B	−	De Vuyst ^61^
			Enterocin 96	−	Henning et al. ^62^
			Enterocin 31	−	Henning et al. ^62^
			Enterocin 1071	−	Martín et al. ^63^
			Enterocin Q	−	Belgacem et al. ^63^
			Mundticin KS	+	Almeida et al. ^64^
			Hiracin JM79	−	Almeida et al. ^64^

+ target gene detected, − target gene not detected.

### Screening for the presence of bacteriocin genes

To assess the presence of bacteriocin-specific genes in L1, L2 and EF, a PCR reaction was performed targeting genes encoding for nisin, lacticin, lactococcin, enterocin, mundticin, and hiracin (Table 1). Amplification reactions were performed with PCR Master Mix (Promega, San Luis Obispo, CA, USA) and the same thermal cycling conditions as described above, modifying the annealing temperature when appropriate. The amplified PCR products were analyzed by 1.2% agarose gel electrophoresis at 100 V for 50 min and bands were visualized with UV light equipment.

### Agar diffusion: evaluation of the antimicrobial effect of BLIS

To assess the potential antimicrobial effect of BLIS from probiotic strains and its possible ability to produce antimicrobial peptides, such as bacteriocins, BLIS sensitivity tests against important bioindicator strains were performed using the agar diffusion test. FIOCRUZ (Rio de Janeiro, RJ, Brazil) provided the pathogen *S.* Typhimurium 5551/16, the Fishing Institute of São Paulo (São Paulo, SP, Brazil) provided the pathogen *S. agalactiae*, whilst the strains *L. monocytogenes* CECT 934, *S. aureus* CECT 237 and *S.* Choleraesuis CECT 724 were acquired from the Spanish Type Culture Collection (CECT) (Valencia, Spain). All isolates were reactivated 24 h before the start of the experiments, followed by pre-inoculum preparation. The optical density (OD_600nm_ 0.8) was determined, the inoculum diluted 100 times (~ 10^6^ CFU/mL), and then incubated according to the initially described growth conditions. After a period of 24 h, the samples were centrifuged at 4470×*g* at 4 °C for 15 min, with 10 mL of the supernatant being removed for subsequent filtration through a 25 μm hydrophilic PVDF membrane (Filtrilo, Colombo, Brazil). The product resulting from this process was the BLIS.

Before testing for antimicrobial activity, the pH of BLIS was adjusted to ~ 6 using NaOH (1 M) and exposed to high temperatures (80 °C/10 min) to stabilize the substance and inactivate possible acids present in the sample. For the agar diffusion test, 1 mL of the inoculum of the pathogens *S.* Choleraesuis and *S.* Typhimurium was added to Petri dishes (90 × 15 mm) containing 10 mL of TSB (Difco, Michigan, USA) and 1 mL of *L. monocytogenes*, *S. aureus* or *S. agalactiae* on BHI agar (Difco, Michigan, USA) in a semi-solid state (supplemented with 0.75% agar). After solidification, 10 µL of the BLIS were pipetted onto the agar, and the plates were incubated for 18 h at 37 °C. Subsequently, inhibition halos were measured with the aid of digital calipers. Antimicrobial activity was expressed as arbitrary units per milliliter (AU/mL) using the formula described below (1), in which π. R^2^ is the area of the inhibition zone (cm^2^) and V is the volume (mL) of BLIS used^28,29^.

1 $$AU/mL=\frac{\pi .R2}{V}$$

### Absorbance microplate reader

An absorbance microplate reader (BioTech, Vermont, USA) was used to assess the mode of action of BLIS against the pathogens tested at different stages of bacterial growth. For this, the BLIS and pathogens were prepared according to the pre-established conditions and incubated in a Microplate Reader (Bioteck Instruments, Vermont, USA) at 37 °C. The OD_600nm_ was determined automatically every hour for 24 h. From this experiment, it was possible not only to confirm the results obtained in the agar diffusion test, but also to determine the stages of bacterial growth that BLIS interfere with. Subsequently, in a sterile 96-well plate (TPP, Trasadingen, Switzerland) all combinations of variables necessary for this analysis were considered, such as positive (BLIS) and negative controls (saline 0.85%), and associations between the BLIS and different pathogens^28,30^.

### Tolerance of isolates to bile salts and low pH

The tolerance to acid pH and bile salts was evaluated based on the methodology described by Tan et al.^31^. L1, L2, and EF previously grown in MRS broth (~ 10^8^ CFU/mL), were centrifuged (4,470 g), washed and resuspended in MRS with pH adjusted to 2, 2.5, 3 and 6 (negative control) with sterile 1 N HCl (Labsynth, Diadema, Brazil). The samples were then incubated at 37 °C, and 1 mL aliquots were taken after 0, 1, 2 and 3 h for CFU counting on MRS 1.5% (w/v) agar.

To evaluate the effect of bile salts, LAB were grown in MRS broth and incubated with bile salts (Sigma-Aldrich, Missouri, USA) at different concentrations (0.1%, 0.2%, 0.3% and the control, without addition) at 37 °C. Aliquots (1 mL) were taken at 0, 2, 4 and 6 h for CFU counting on MRS 1.5% (w/v) agar plates.

### Tolerance of BLIS to low pH, high temperatures and proteolytic enzymes

To verify the stability of BLIS against different temperatures and pH, the method described by Todorov and Dicks^32^ was used. To this end, BLIS were subjected to heat treatments (30, 50, 70 or 90 °C for 1 h; 121 °C for 15 min) and pH treatments adjusted to pH 2, 4, 6, 8 or 10 with 1 N NaOH and HCl; Labsynth, Diadema, Brazil) at 30 °C for 1 h. To evaluate the proteinaceous nature of BLIS, samples were subjected to 1% (w/v) trypsin, pepsin, papain or pancreatin (Inlab, Alamar Tecno Científica Ltda, São Paulo, Brazil) and incubated at 30 °C for 2 h. After this period, the stability of BLIS was verified using the diffusion agar technique against *L. monocytogenes*.

### Hemolytic activity

The production capacity of the extracellular protein hemolysin was evaluated in Petri dishes containing BHI agar supplemented with 5% sheep's blood. After preparing the inoculum, the isolates were spread on the surface of the sheep's blood agar and incubated according to the pre-established growth conditions. The activity of hemolytic hemolysin protein was confirmed by the formation of different types of halos, whose interpretation was performed by their coloring: α-hemolysin when there were greenish areas around the colonies, β-hemolysin when the zones were light-colored, and γ-hemolysin in the absence of such zones^33^.

### Gelatinase production

For the gelatinase production test, the inoculum was cultivated on the surface of Petri dishes containing BHI supplemented with skimmed milk (1.5%) and incubated according to the respective growth conditions described above. According to Tan et al.^31^, a clear halo around the colony indicates a positive result for gelatinase production.

### Coexistence test

This test investigates the possibility of co-cultivation between the three probiotic bacteria evaluated in this study. The tests were carried out according to the method described by Guo et al.^34^. Specifically, the bacteria were grown in their respective growth conditions for 24 h, and then samples were streaked perpendicularly to each other on the surface of plates containing 1.5% MRS (w/v) agar. After a 24-h incubation period, plates were examined for possible antagonistic effects.

### Antibiotic resistance

Antibiotics of clinical importance were used, including vancomycin (30 μg), clindamycin (2 μg), streptomycin (10 μg), gentamicin (30 μg), chloramphenicol (30 μg), rifampicin (5 μg) and ampicillin (10 μg) (all provided by LABORCLIN, São Paulo, Brazil) loaded onto disks. Therefore, isolates were reactivated in the conditions mentioned above and, after 24 h of cultivation, bacterial growth at OD_600nm_ was determined and adjusted to 0.8. Finally, the samples were streaked on the surface of a Petri dish containing Mueller Hinton agar (Difco, Michigan, USA) and, after drying, the antibiotic-containing disks were added to the plates. Following incubation at 37 °C for 24 h, the presence or absence of inhibition halos around the disks was interpreted^35^.

### Adherence to intestinal epithelial cells

The method described by Jensen et al.^36^ was used, with minor changes. For this, DMEM medium (Vitrocell Embriolife, Campinas, Brazil) was added to 24-well culture plates with 2105 human colon adenocarcinoma cells (Caco-2; ATCC HTB-37, Manassas, USA) with low content glucose, 20% (v/v) fetal bovine serum (Vitrocell Embriolife, Campinas, Brazil) and 100 U/mL antibiotics (penicillin/streptomycin) (Sigma-Aldrich, St. Louis, USA). Then, the plates were incubated at 37 °C (humidified atmosphere, 5% CO_2_ and 95% air) for three days, until the appropriate growth point was reached. To perform the adhesion test, the isolated bacteria were grown for 24 h in suitable conditions and centrifuged (10,000×*g* for 10 min), and the pellet was resuspended in DMSO medium (without antibiotics). The monolayer formed by the growth of Caco-2 cells was washed twice with PBS before the start of the adhesion test, so that there was complete removal of the antibiotic used in the cell growth medium.

Thus, 1 mL of each bacterial culture (10^7^ CFU/mL) was transferred individually to the wells and the plates were incubated at 37 °C for 1, 2 or 4 h, to optimize the assay. Subsequently, the cell monolayers were washed twice (PBS) to remove bacteria that were unable to adhere, and lysis of the monolayer was performed by adding PBS with 0.1% Triton-X100 (Sigma-Aldrich, St. Louis, USA). The resulting suspension (viable adherent bacteria) was diluted in different concentrations and incubated in MRS medium (pouring plate method) for 48 h. At the end of the experiment, the number of CFU/mL was determined and results were expressed as a percentage. Additionally, the ratio between the number of bacterial cells that remained adhered to the monolayer and the total number of bacterial cells added was measured.

### Statistical analysis

The mean and standard deviation were used to express the results. The counts of viable bacteria were transformed into log values. The values in the tolerance test were compared using the software Statistica 12.0 (TIBCO, Palo Alto, CA, USA) applying the Tukey test with a level of significance *p* < 0.05.

## Results

### Isolation and identification by MALDI-TOF and 16S rRNA sequencing

A substantial number of CFU isolated from the cecum content of rainbow trout (*Oncorhynchus mykiss*) and starfish (order *Forcipulatida*) were observed. Subsequently, the isolated bacteria were collected and used in biochemical and morphological identification tests. All isolates belonging to the LAB group were selected for the next stages of this study and the bacteriocin-like inhibitory substances (BLIS) of each one were evaluated for their antimicrobial effect against important pathogens of fish and food. From rainbow trout samples, two isolates identified via MALDI-TOF as *Lactococcus lactis* (L1) and another as *Lactococcus garvieae* (L2), and one isolate from starfish identified as *Enterococcus faecium* 135 (EF) were selected for further molecular identification. The results obtained using the 16S rRNA method confirmed the previous data obtained by MALDI-TOF for isolates L1 and EF; however, the molecular analysis indicated that isolate L2 is *L. lactis*. Sequences generated in this study were deposited at GenBank (NCBI) under accession numbers MZ926851, MZ735396 and MZ926852, respectively.

### Tolerance of the isolates to low pH and bile salts

**Figure 1:**
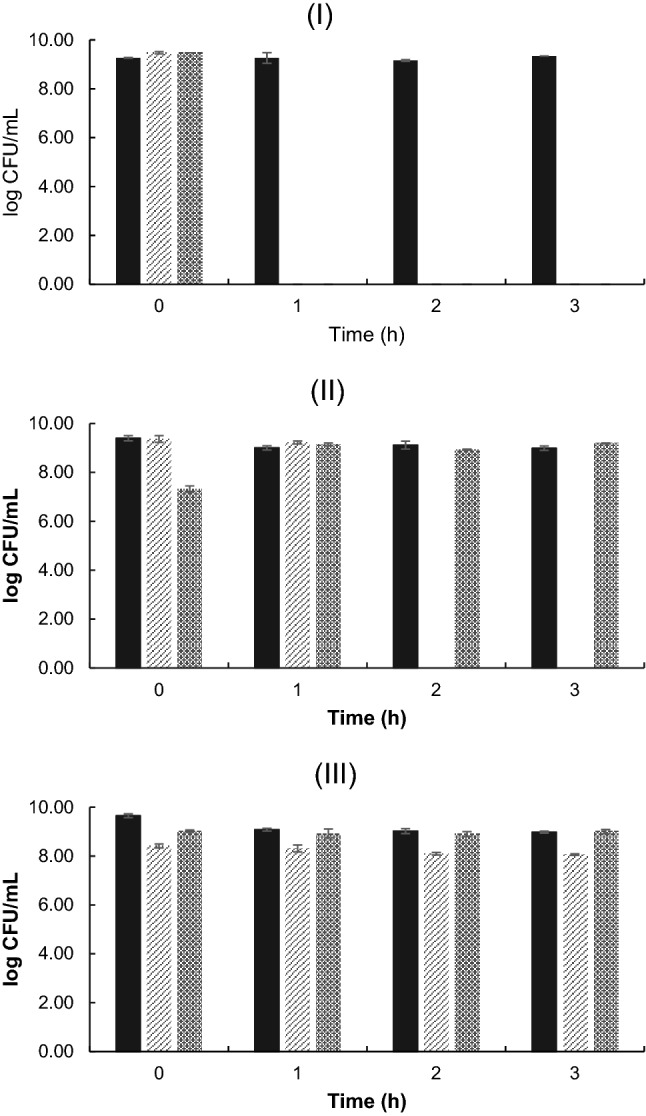
Tolerance of L1 (**I**), L2 (**II**) and EF (**III**), to pH 3 () and 0.3% bile salts (). Strains without treatment of acid and bile salts were used as controls (filled black square). Bars represent means ± standard deviation, n = 3.

To assess the resistance of the isolates to environments that reflect the adverse conditions of the gastrointestinal tract, they were exposed to different pH and concentrations of bile salts (Fig. 1). In the test of tolerance to different pH (Table S1), L1 was able to grow only in control conditions (pH 6). The same behavior was observed in the test with bile salts, where after 1 h of incubation there was no growth of L1 in any of the concentrations tested. Therefore, L1 was sensitive to low pH and high concentrations of bile salts, indicating that it must be protected by, for example, microencapsulation, if it is to be used as a probiotic. In contrast, the L2 isolate grew until 1 h of incubation at pH 3, and no negative effect was observed in the test with bile salts, with good growth observed in all concentrations tested. Finally, EF was resistant to low pH and bile salts during all evaluated periods (Table S2), with the test data with 0.3% bile similar to the results in control conditions.

### Hemolysin and gelatinase virulence factors

The capacity of the isolates to produce the extracellular proteins gelatinase and hemolysin was evaluated. None showed α or β-hemolytic profiles and there was also no gelatinolytic activity, since the physical properties of the agar remained unchanged.

### Antibiotic susceptibility testing

The susceptibility of isolates to the main antimicrobials of clinical interest was evaluated. In this sense, the three isolates possessed different sensitivity profiles, as observed from the measurement of inhibition halos when cultivated with the different antibiotics tested. Of note is that L1 was especially sensitive to ampicillin and clindamycin, and L2 and EF to clindamycin and rifampicin. When gentamicin was tested against EF, it was observed that the isolate is not very sensitive; however, its degree of resistance was considered low^37^, so it could not be defined as resistant (Table 2).

**Table 2:** Sensitivity of isolates to antibiotics by diffusion in agar.

Isolated probiotic strains	Antibiotic			
	Name	Disc concentration (μg)	Inhibition zone (mm)	Results *
L1	Ampicillin	10	64.30	S
	Vancomycin	30	39.92	S
	Streptomycin	10	32.88	S
	Gentamicin	10	45.35	S
	Rifampicin	5	18.21	S
	Chloramphenicol	30	52.75	S
	Clindamycin	2	60.03	S
L2	Ampicillin	10	37.08	S
	Vancomycin	30	35.42	S
	Streptomycin	10	15.54	S
	Gentamicin	10	24.60	S
	Rifampicin	5	46.48	S
	Chloramphenicol	30	44.46	S
	Clindamycin	2	48.84	S
EF	Ampicillin	10	25.50	S
	Vancomycin	30	23.00	S
	Streptomycin	10	8.50	R
	Gentamicin	10	12.50	MS
	Rifampicin	5	30.50	S
	Chloramphenicol	30	29.50	S
	Clindamycin	2	31.50	S

*S* susceptible, *R* resistant, *MS* mostly resistant. *Charteris et al.^37^.

### Adhesion test to intestinal cells

**Figure 2:**
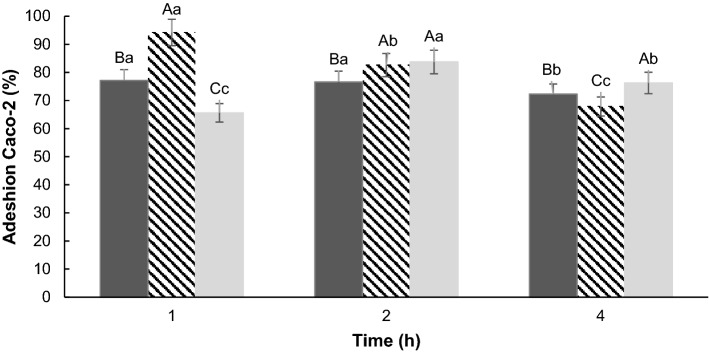
Adhesion (%) of L1 (filled black square), L2 () and EF (filled gray square) to Caco-2 cells, after 1, 2 and 4 h of incubation. Different uppercase letters indicate statistically significant differences for all cultures taken at the same time (P < 0.005). Different lowercase letters indicate statistically significant differences for the same strain at different timepoints (P < 0.005). Bars represent means ± standard deviation, n = 3.

All three isolates adhered to Caco-2 cells (Fig. 2). After the first hour of the experiment, L2 presented an adhesion of 94.2%, L1 77.1% and EF 65.6%. In the second hour, the adhesion percentages of L2 and EF were statistically similar (83%, P > 0.005), whilst L1 adhesion fell only marginally (76.6%, P > 0.005) compared to the first hour. It was observed that after the fourth hour of the experiment, all isolates tested suffered a reduction in adherence, ranging from 67.9% (L2) to 76.2% (EF). L1 possessed the most stable adherence over the time course of the assay. For L1 and L2, only one hour was necessary for the cells to adhere to the Caco-2 cells, whilst the best adherence of EF was obtained after 2 h. With the high percentages of adherent cells, we conclude that if these isolates were administered to a host, they would probably adhere to intestinal cells, and exert a probiotic effect.

### Coexistence test

**Figure 3:**
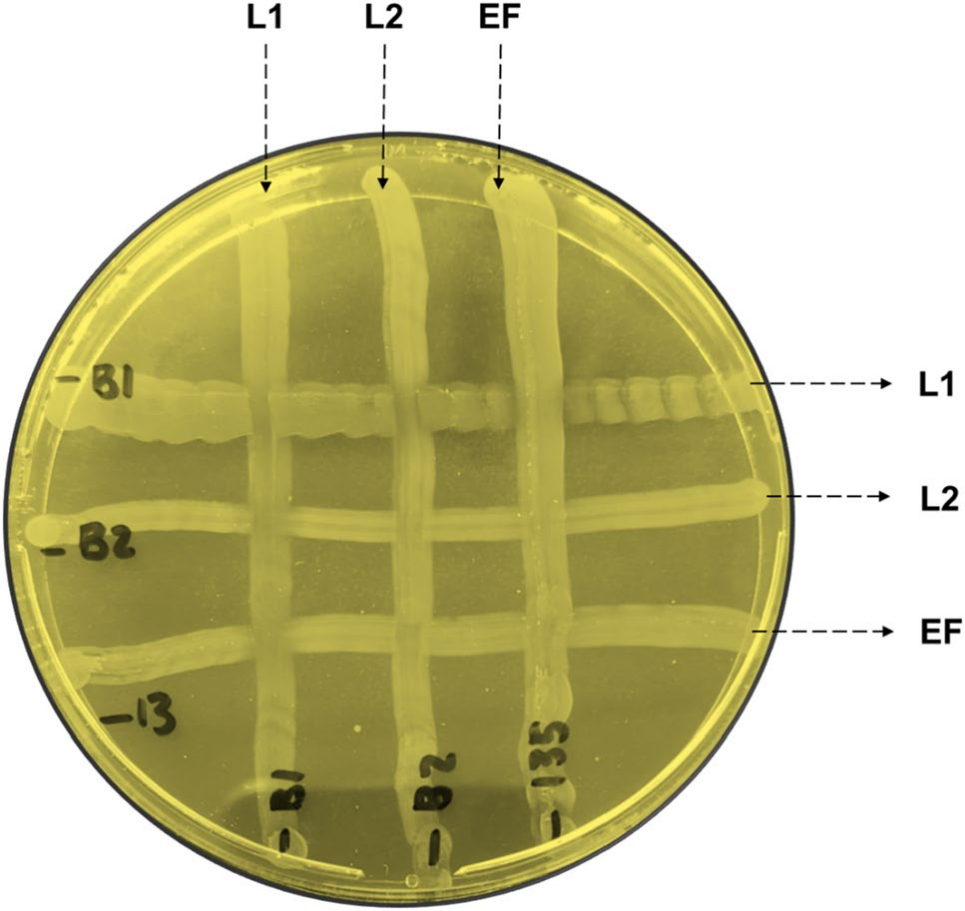
Coexistence test between isolates *L. lactis* (L1 and L2) and *E. faecium* (EF). No antagonist effects were observed.

After plating L1, L2 and EF in crossed lines, plates were incubated for 48 h at 37 °C. At the end of the experiment, it was observed that there was a substantial growth of all isolates tested and no antagonistic effects were evident (Fig. 3).

### Bacteriostatic effect of BLIS and interference with different growth stages

To assess the antimicrobial potential of BLIS produced by isolates and their possible capacity to produce bacteriocins, BLIS sensitivity tests against important pathogens were performed. After the incubation period, the formation of inhibition halos was observed. These were measured, and the antimicrobial effect of BLIS was determined by quantifying the area of the halo, considering the amount of BLIS used (Table 3). The BLIS of L1 had a good inhibitory effect against *Listeria monocytogenes* and *Staphylococcus aureus*, L2 against *L. monocytogenes*, *S. agalactiae*, *S. aureus* and *Salmonella* Choleraesuis and EF against *L. monocytogenes and S.* Choleraesuis. Furthermore, the quantification of BLIS produced by the isolates revealed that L2 was the largest producer, particularly inhibiting the pathogens *L. monocytogenes* and *S. aureus*. None of the three isolates was able to inhibit the growth of *Salmonella* Typhimurium in this agar diffusion test.

**Table 3:** Average diameter (cm) and quantification (AU/mL) of the BLIS inhibition halos against pathogens.

Bioindicator strains	BLIS of L1		BLIS of L2		BLIS of EF	
	Inhibition zone (cm)	Quant. (AU/mL)	Inhibition zone (cm)	Quant. (AU/mL)	Inhibition zone (cm)	Quant. (AU/mL)
*S. agalactiae*	1.300	132.660	1.460	167.420	–	–
*L. monocytogenes*	1.035	162.338	1.629	255.596	2.282	408.790
*S. aureus*	1.025	160.768	1.014	159.198	–	–
*S. Choleraesuis*	–	–	0.898	140.986	1.263	125.220
*S. Typhimurium*	–	–	–	–	–	–

“–” no inhibition.

**Figure 4:**
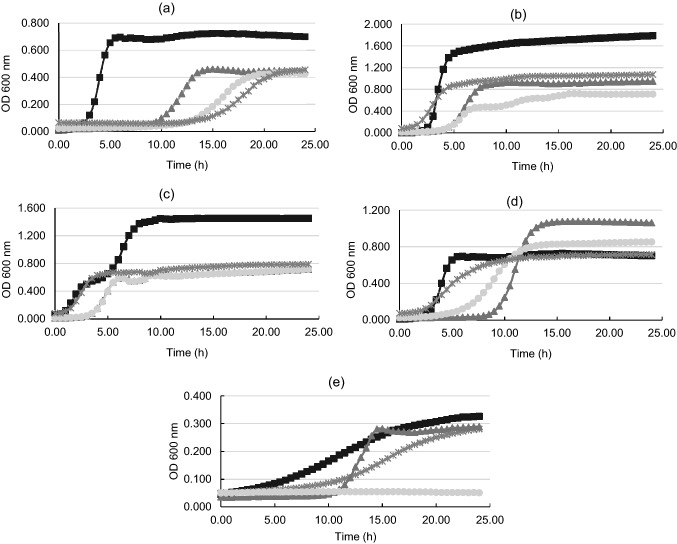
Antimicrobial activity of BLIS produced by L1 (filled black triangle), L2 (filled gray circle), and EF (cross symbol) against the pathogens *L. monocytogenes* (**a**), *S. Choleraesnius* (**b**), *S. Typhimurium* (**c**), S. *aureus* (**d**), and S. *agalactae* (**e**). Assays performed with positive controls (filled black square). The results are represented as an average of three readings.

These preliminary findings were corroborated by using a microplate reader, as a means of assessing BLIS mode of action against the tested pathogens. From this experiment, it was possible not only to confirm the positive results obtained in the agar diffusion test, but also to pinpoint the specific bacterial growth stage that was affected by BLIS. In general, it was observed that there was interference by the BLIS of all isolates in all growth phases of the pathogens, especially in the delay of the LAG phase and the early stages of the LOG phase, which is equivalent to the full exponential multiplication phase of microorganisms. In this experiment, *L. monocytogenes* was the most sensitive pathogen and the BLIS produced by L2 was the most potent (Fig. 4).

In the test with *L. monocytogenes* (Fig. 4a), BLIS of all isolates delayed the initial growth phases. Notably, the BLIS of EF and L2 delayed the end of the LAG phase of *L. monocytogenes* for up to 13 h/OD_600nm_ 0.07 and 12 h/OD_600nm_ 0.06, respectively, longer than the control (2h50/OD_600nm_ 0.08). In the group treated with the BLIS of EF, *L. monocytogenes* reached the beginning of the stationary phase at 21h50/OD_600nm_ 0.45 compared to 5h50/OD_600nm_ 0.70 in the control group. When general pathogen growth data were compared with those of the control, it was noted that the BLIS of the isolates effectively slowed pathogen growth, an important indication of their potential use and of the possible presence of molecules with antimicrobial effects similar to bacteriocins.

Challenges with *S. *Choleraesuis and *S. *Typhimurium had similar results in this test. When exposed to BLIS of L1 and L2, the time needed by *S. *Choleraesuis to reach the end of the LAG and LOG phase increased (Fig. 4b). The BLIS of EF influenced the growth of both pathogens similar to the control, but it was able to maintain an OD_600nm_ of 1.0. However, the BLIS of L2 had the most potent antimicrobial effect, maintaining not only the microbial population at levels below the control amounts, but also delaying the end of the LOG phase from 5 h/OD_600nm_ 1.4 to 16 h/OD_600nm_ 0.71 in the treated group.

Unlike the agar diffusion test results, BLIS derived from the three isolates inhibited *S. *Typhimurium growth (Fig. 4c). EF was able to reduce the OD_600nm_ by half when compared to the control, and L1 and L2 had similar effects of prolonging the LAG phase. Once again, a significant reduction in absorbance and an increase in the time of the LAG and LOG phase of bacterial growth, compared to the control, was observed. L2 proved to be the most potent; in the treated group, the stationary phase was reached at 6 h/OD_600nm_ 0.58 versus 9 h/OD_600nm_ 1.4 in the control group. Despite this, BLIS of L2 exerted its bacteriostatic effect throughout the period, limiting growth to just half of the OD_600nm_ seen in the control group.

The interference in the microbial growth phases occurred differently in tests with *S. aureus* (Fig. 4d). When exposed to BLIS, especially from L1, more time was required for *S. aureus* to reach the end of the LAG phase (9 h/OD_600nm_ 0.09, against 3 h/OD_600nm_ 0.12 in the control). However, growth superior to that of the control was observed, a finding repeated in multiple independent assays. This may be because after the delay in the start of the exponential phase, there may have been an increase in the consumption of substrates present in the medium; alternatively, BLIS might boost growth when these biomolecules lose their inhibitory effect. Further investigations are thus needed to clarify the causes.

Regarding the test with *S. agalactiae* (Fig. 4e), all the different BLIS used were able to prolong the LAG phase. Of special note is that the bactericidal effect of the BLIS of L2 prevented pathogen growth, as observed by the maintenance of OD_600nm_ below 0.1 throughout the experiment.

### Tolerance of BLIS to low pH and high temperatures

The tolerance of BLIS to low pH and high temperatures was also investigated. In this sense, the cell-free supernatant of the isolates was recovered and subjected to different pH (2, 4, 6, 8 and 10) and temperature (30, 50, 70, 90 and 120 °C) treatments and then tested against *L. monocytogenes* (Figs. S1 and S2)*.* It was observed that the BLIS of L1 and L2 maintained their activity against the pathogen up to 70 °C, while EF maintained its activity up to 90º C. In the exposure test to different pH, none of the BLIS lost activity at any of the different pH values tested.

### Assessment of the protein nature of BLIS

An important step in the characterization of BLIS is the use of proteolytic enzymes to assess their possible protein nature. As already described, since bacteriocins are characterized as antimicrobial peptides, it is expected that there is a loss of antimicrobial activity after treatment with enzymes such as trypsin, pancreatin, papain and pepsin (Fig. S3). In such assays, when compared to the control group, EF BLIS had a total loss of inhibitory activity after incubation with all enzymes tested (Table 4). In turn, L1 and L2 BLIS had a considerable loss of inhibitory activity after treatment with all 4 enzymes. These data strongly suggest the presence of protein molecules with antimicrobial activity in BLIS from all three aquatic isolates.

**Table 4:** Effect of enzymatic treatment, pH and temperature on the stability of the BLIS produced by *L. lactis* (L1 and L2), and *E. faecium* 135 (EF).

Treatment	Inhibition zone *		
	L1	L2	EF
Control	+++	+++	+++
**Enzymatic treatment**			
Trypsin	++	++	−
Pepsin	+	+	−
Papain	+	+	−
Pancreatin	+	+	−
**pH resistance**			
2, 4, 6, 8 and 10 for 1 h	+++	+++	+++
**Heat treatment**			
30, 50, 70 or 90 °C for 1 h	+++	+++	+++
120 °C for 15 min	−	−	−

*(+++) > 12 mm, (++) 10–11.99 mm, (+) 8–9.99 mm, and (–) did not show inhibition zone. The bioindicator strain used to evaluate antimicrobial activity was *Listeria monocytogenes* CECT 934. Control: BLIS without any treatment. The concentration of the enzymes used in the experiments was 1% (w/v).

### Presence of genes for different bacteriocins

As a preliminary approach, a study was carried out to detect the main bacteriocins that have been described in the literature in recent years for bacteria of the genus *Lactococcus* and *Enterococcus*. Primers were designed and synthesized to amplify the most well-studied bacteriocins of these genera, which were subsequently used for amplification in PCR reactions. The PCR amplicons were analyzed, revealing the presence of promising amplicons (Fig. S4) for Nisin in L1 and L2 and for different Enterocins A, B and P, and for Mundticin KS in EF (Table 1).

## Discussion

In this study, we demonstrated that the intestinal tracts of two aquatic animals are an important source of probiotic bacteria with bacteriocinogenic potential^38^. These results corroborate the data of Sarika et al.^39^, where the authors report that a strain of *L. lactis* PSY2 isolated from marine perch (*Perca flavescens*) had a bacteriocinogenic profile and a significant antimicrobial effect against several Gram-negative and Gram-positive bacteria, such as *L. monocytogenes* and *S. aureus*. The authors also emphasize that such a bacteriocinogenic profile can assist in food preservation; in tests carried out with the strain there was an increase of more than 21 days of shelf-life, useful for the preservation of high-value seafoods. Thus, concerning our study, it is important to emphasize that once the antimicrobial potential of the BLIS identified in the three isolates has been demonstrated, specific studies will be carried out to evaluate their possible use in seafood preservation.

After confirming the presence of genes for bacteriocins in all isolates (such as Nisin and Enterocin) and the loss of BLIS activity after enzymatic treatment, their bacteriocinogenic potential should be evaluated further. Indeed, the preliminary tests demonstrated that the L2 and EF isolates from rainbow trout and starfish, respectively, are not only bacteriocin producers, but also have substantial probiotic potential, as they can resist pH 3 and various concentrations of bile salts. In this study, among the pathogens analyzed, *L. monocytogenes, S.* Choleraesuis and *S.* Typhimurium were the most sensitive to the bacteriostatic effect of the isolates. The BLIS of L2 had the best results in the inhibition tests, including a bacteriostatic effect against *S. agalactiae*.

The *Salmonella* pathogen is a major concern for the food industry, as it is transmitted through contaminated food and water. In recent years, probiotic bacteria have been studied for the control of the pathogen with promising results^40^. The preliminary inhibition results observed in our study need to be further evaluated. Nevertheless, they are promising, as they indicate that bacteriocins could be used as a possible non-chemical containment strategy for these pathogens. In a similar survey, Sahnouni et al.^41^ investigated the antagonistic effect of 38 LAB isolates against several pathogens, including *Salmonella* sp. The BLIS tested were found to be ineffective against Gram-negative bacteria such as *Salmonella* sp. and *Escherichia coli*, compared to the others. However, in an in vivo study, Mulaw et al.^42^ observed a different result. These authors tested a mix of probiotic bacteria (*Lactobacillus plantarum* K132, *Lactobacillus paracasei* K114 and *L. lactis* E124) against infection by *S.* Typhimurium DT104 in mice. They observed that, compared to the control group, treatment with a mix of probiotics led to a reduction in *S.* Typhimurium DT104 counts in feces and the survival rate was significantly higher.

In the test of tolerance to low pH and different concentrations of bile salts, isolates EF and L2 had the best results, with EF resisting all ranges of pH and bile salts tested. As in our study, Yerlikaya^43^ evaluated isolated probiotic bacteria in order to select strains for the production of functional foods. During the characterization phase of isolated *L. lactis* strains, the researchers evaluated their ability to resist bile salts and found that none of the tested strains managed to grow in their presence, an important indicator of the high sensitivity of the genus *Lactococcus* to such substances. In turn, Jawan et al.^43^ also evaluated the susceptibility of *L. lactis* Gh1 to these factors and found that the strain was tolerant to pH 3 and bile salts at a concentration of 0.3%, indicating that resistance against these factors is strain-specific. Moreover, Dowdell et al.^44^ demonstrated the ability of *E. faecium* and *L. lactis* to survive a simulation of adverse conditions in the gastrointestinal tract. The results were similar to those present in our study, and the authors also demonstrated the superior ability of EF to survive acidic environments when compared to *L. lactis*.

In this study, we have emulated the gastrointestinal tract conditions similar to the ones observed in the host, such as high acidity and the presence of bile salts, where probiotic strains can grow and survive. By demonstrating resistance in these tests, the probiotic strain becomes an important candidate to demonstrate its potential in in vivo studies^45^. Thus, Fahim et al.^46^ state that a viable alternative would be the use of microencapsulation to increase cell viability. According to the authors, the use of microencapsulation with alginate in association with chitosan offers protection to both the probiotic and biomolecules in the passage through the gastrointestinal tract. Other studies, such as those of Rodklongtan et al.^47^, Song et al.^48^ and Zohri et al.^49^, also report increased cell viability after using different microencapsulation techniques.

Considering that one of the longer term objectives of the present work is the biotechnological application of isolated bacteria and their biomolecules in the formulation of, for example, functional foods, the expression of hemolysin and gelatinase virulence factors in the isolates needed to be investigated. This is because the presence of microorganisms with such characteristics in food matrices is a problem, as these virulence factors may be associated with the development of serious diseases and death^31,33^. Therefore, the absence of expression of such virulence factors in this study is encouraging, although the presence of other virulence genes also needs to be evaluated before performing experiments in vivo.

In the same sense, one of the most undesirable characteristics of a probiotic microorganism is the ability to withstand exposure to antibiotics. In our study, none of the isolates showed resistance to the antibiotics tested, all of which are of clinical importance. Therefore, our results are of great importance and reflect what has also been previously described by other studies with LAB^33,50,51^.

After evaluating the expression of these important virulence factors, future work should focus on the possibility of using the isolates in a probiotic mixture. Indeed, Mariam et al.^52^ isolated probiotic strains belonging to the LAB group and, after several tests, raised an important issue. Specifically, according to the authors, co-culture in mixtures was not only possible but also increased BLIS antimicrobial action, which started to inhibit pathogenic bacteria such as *L. monocytogenes* (a microorganism that can resist common food preservation methods) more effectively, thus reducing cell count to much lower levels than the control group. For this reason, the authors encourage studies with new probiotic strains to assess their interaction in mixed cultures.

In the experiment with Caco-2 cells, the percentage of adherence was high for all isolates tested (> 70%), a finding which is encouraging for future in vivo studies. Although promising, the high adhesion potential of *L. lactis* and *E. faecium* is well described in the literature. Nascimento et al.^53^ and Downdell et al.^44^ carried out similar studies and obtained good adherence percentages, but lower than those observed in our study. Vasiee^54^, in turn, evaluated the adherence potential of the recombinant strain *L. lactis* NZ1330 to Caco-2 cells and its antagonistic effect on *E. coli*. In the end, a good adhesion potential and ability to compete and prevent the adhesion of *E. coli* to Caco-2 cells were observed. Furthermore, He et al.^55^ demonstrated the ability of *E. faecium* WEFA23 to compete and inhibit (> 50%) the adherence of *L. monocytogenes* and *S.* Typhimurium to Caco-2 cells.

The results observed in this study suggest that using bacteriocinogenic probiotic strains as a food supplement might be a feasible strategy for the control of infectious diseases. The increase in recently published studies in the area demonstrating the beneficial effects on health and disease resistance after supplementation with probiotics reveals the great scientific potential of this segment^56,57^.

In summary, based on the promising results obtained in this study, a bacteriocin purification study, as well as an evaluation of the protective potential of microencapsulation on the isolates and the individual and concomitant (mix) probiotic effect in an in vivo test, will be performed.

## Conclusions

The aquatic environment proved to be an important source of bacteriocinogenic probiotic bacteria. All isolates evaluated in this study harbor genes for bacteriocins, showed antimicrobial activity against important fish and food pathogens, were sensitive to all antibiotics tested, had a high rate of adherence to Caco-2 cells and did not express hemolysin and gelatinase virulence factors. It was shown that isolates L1 and L2 from rainbow trout were not able to resist low pH. However, isolates L2 and EF (from starfish) demonstrated good resistance to the action of bile salts, and EF was also resistant to pH 2.5 and 3. For this reason, future tests to evaluate the protective effect of microencapsulation on the viability of the isolates and their effect on an animal model will be carried out. There is no doubt that the new discoveries in the field of probiotics will bring countless changes in this area of study, which will result in ever higher quality foods and consumer health, whilst lowering impacts on nature. One of the main advances brought about by research with individual and mixtures of probiotics, is the gradual replacement of antibiotics, a decreased in new episodes of microbial resistance and better responses to production diseases, commonly treated with chemicals or antibiotics.

## Supplementary Information

Supplementary Information.
