# Supplementary material for: Bacteriocinogenic probiotic bacteria isolated from an aquatic environment inhibit the growth of food and fish pathogens
Source: Sci Rep. 2022 Apr 1;12:5530. doi: 10.1038/s41598-022-09263-0 (PMC8975912; doi:10.1038/s41598-022-09263-0)
Supplement: Supplementary file 1 — Supplementary Information. [file 41598_2022_9263_MOESM1_ESM.docx]

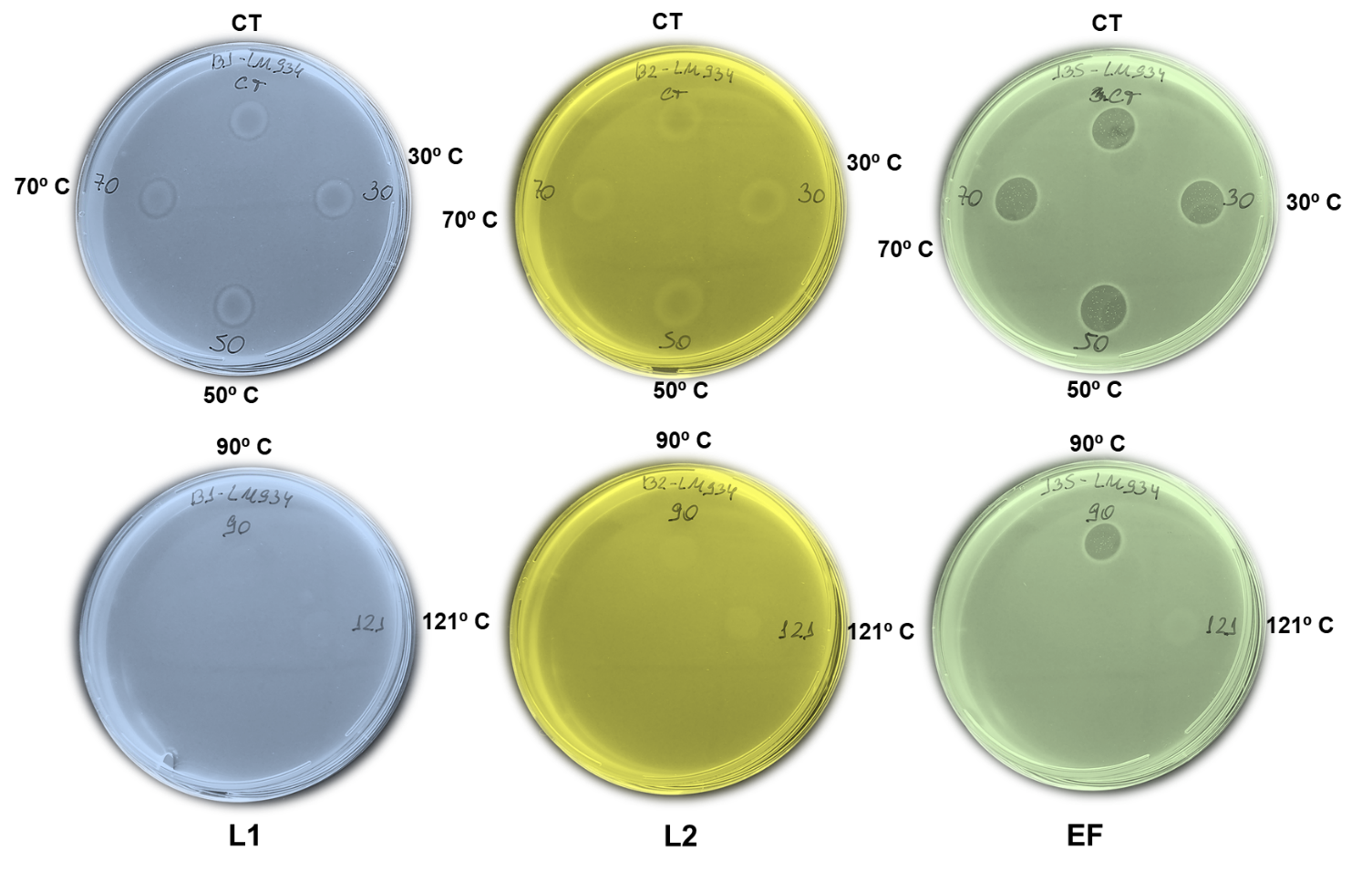


**Figure S1.** Exposure of BLIS produced by isolates to different temperatures. CT= control (BLIS without the temperature treatment applied in the other samples).


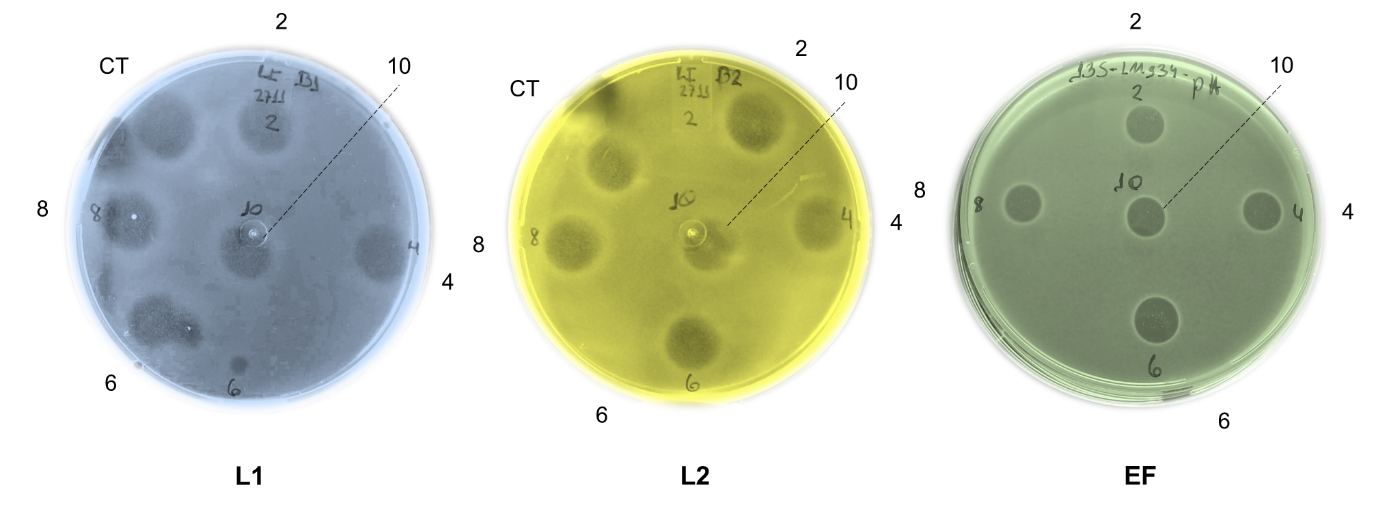


**Figure S2.** Exposure of BLIS produced by isolates at different pHs. CT= control (BLIS without the pH treatment).


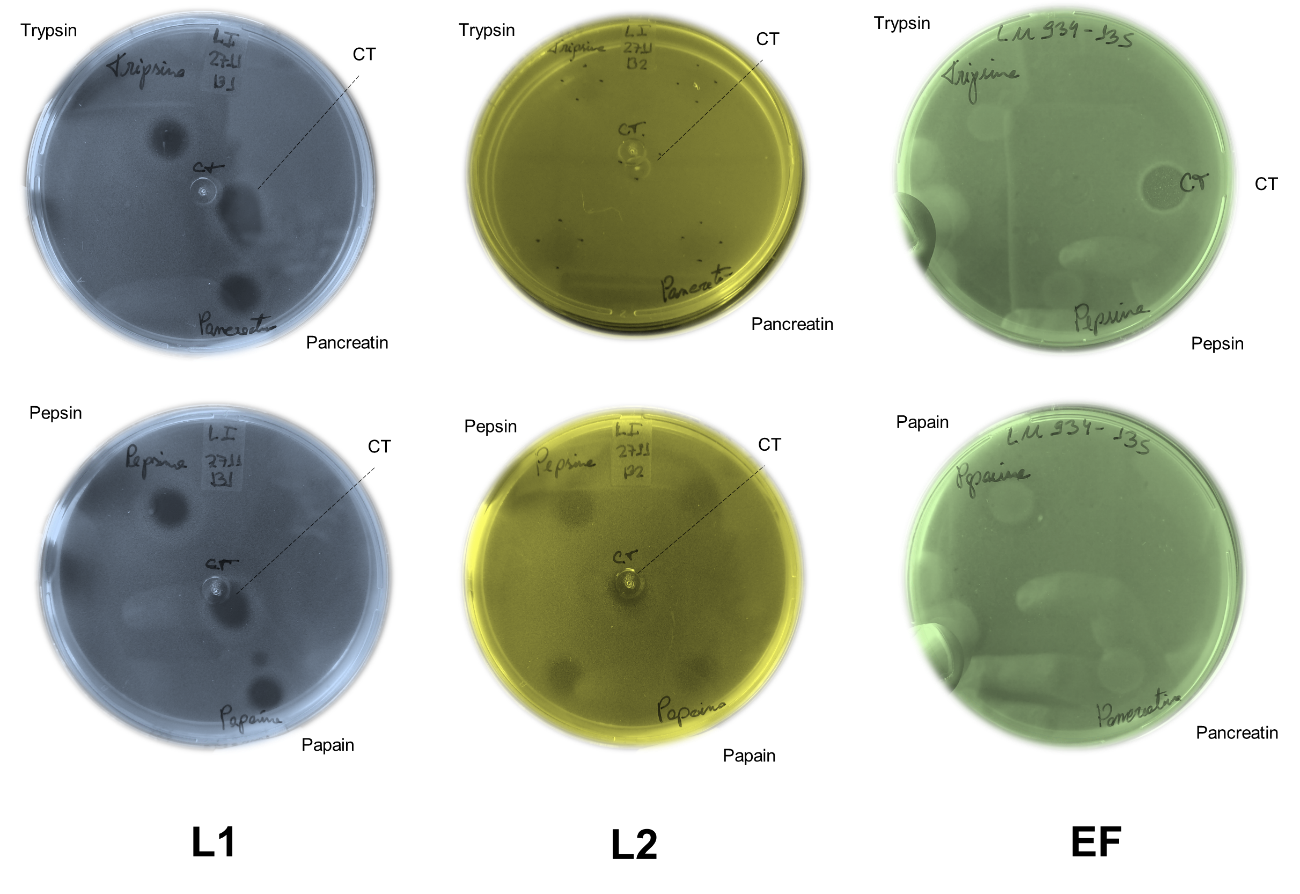


**Figure S3.** Exposure of BLIS produced by isolates to different proteolytic enzymes. CT= control (BLIS without the enzymatic treatment).

M

ENT A

ENT B

ET P

MUN

C -


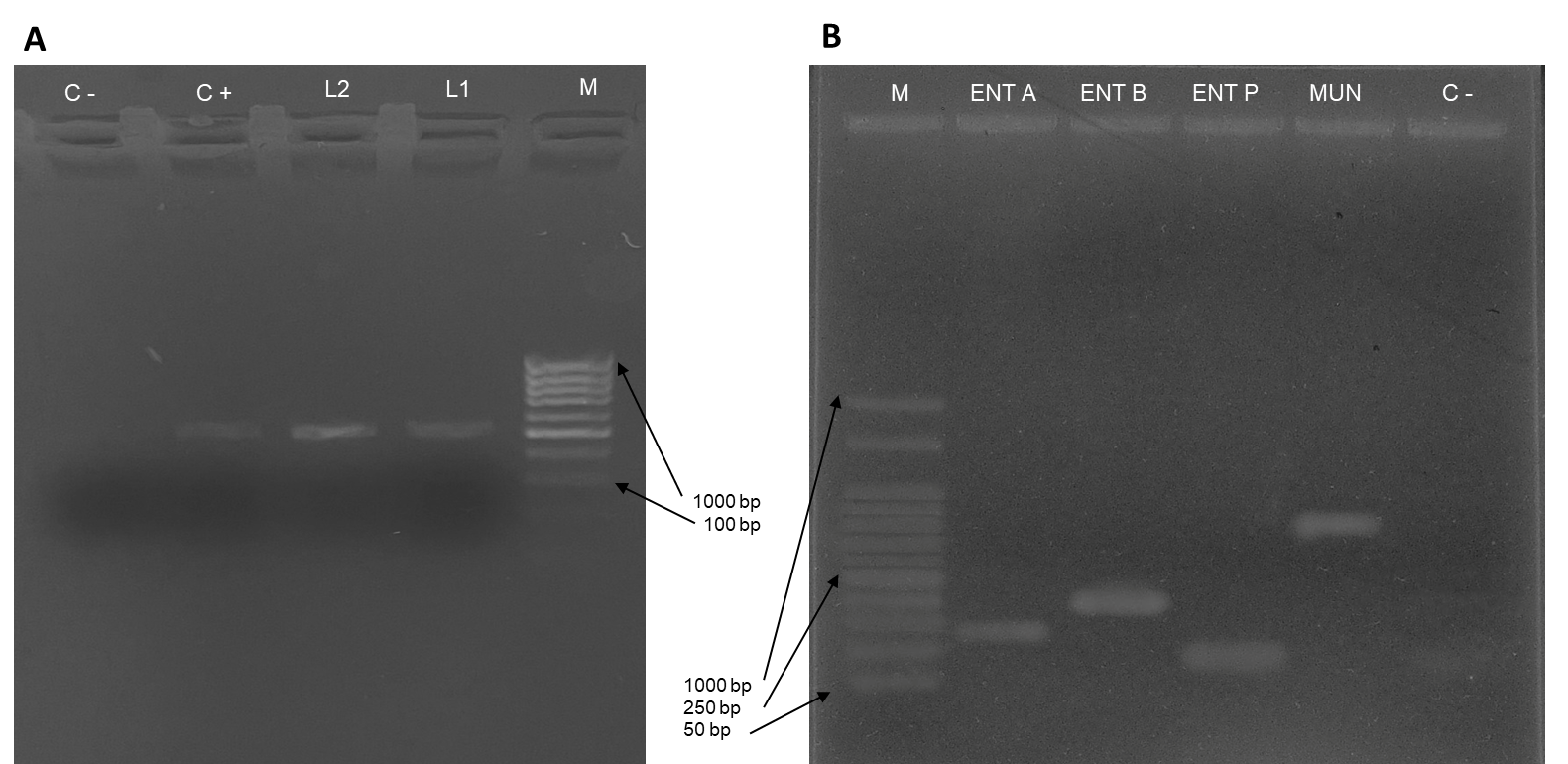


**Figure S4.** Screening for presence of bacteriocin genes in *L. lactis* L1 – L2 (A) and *E, faecium* strains (B). Lane M: 1 kb DNA markers; Lane C + and C -: products of positive and negative control PCR reactions, respectively. Lane L2 and L1: PCR amplification products of the nisin gene of *L. lactis* L1 and L2; Lane ENT A, ENT B, ENT P, and MUN: PCR amplification products of bacteriocin genes of *E. faecium*.

**Table S1.** Effect of acids (pH 2, 2.5 and 3) on the viability (log CFU/mL) of *L. lactis* (L1 and L2) and *E. faecium* (EF).

| **Strain** | **pH** | **Time** | | | |
| --- | --- | --- | --- | --- | --- |
|  |  | 0h | 1h | 2h | 3h |
| L1 | Control | 9.27 ± 0.01 ^Aa^ | 9.26 ± 0.22 ^Aa^ | 9.15 ± 0.03 ^Aa^ | 9.34 ± 0.00 ^Aa^ |
|  | pH 2 | 7.45 ± 0.15 ^D^ | - | - | - |
|  | pH 2.5 | 8.78 ± 0.18 ^C^ | - | - | - |
|  | pH 3 | 9.47 ± 0.04 ^A^ | - | - | - |
|  |  |  |  |  |  |
| L2 | Control | 9.15 ± 0.11 ^Aa^ | 9.09 ± 0.09 ^Aa^ | 9.12 ± 0.16 ^Aa^ | 8.99 ± 0.09 ^Ba^ |
|  | pH 2 | 7.95 ± 0.05 ^E^ | - | - | - |
|  | pH 2.5 | 9.14 ± 0.06 ^A^ | - | - | - |
|  | pH 3 | 9.31 ± 0.14 ^Aa^ | 9.14 ± 0.06 ^Aa^ | - | - |
|  |  |  |  |  |  |
| EF | Control | 8.45 ± 0.08 ^Ba^ | 8.25 ± 0.05 ^Bb^ | 8.46 ± 0.10 ^Ba^ | 8.59 ± 0.04 ^Ca^ |
|  | pH 2 | 8.59 ± 0.19 ^BC^ | - | - | - |
|  | pH 2.5 | 8.69 ± 0.09 ^BCa^ | 6.78 ± 0.35 ^Cb^ | 5.03 ± 0.10 ^Dc^ | 3.69 ± 0.20 ^Ed^ |
|  | pH 3 | 8.41 ± 0.08 ^Ba^ | 8.31 ± 0.14 ^Ba^ | 8.09 ± 0.05 ^Cb^ | 8.06 ± 0.03 ^Db^ |

The results are expressed as means ± standard deviations, n = 3; (-) indicates that the counts
were < 100 CFU/mL. Different uppercase letters in the same column mean statistically different values according to the Tukey’s test (P < 0.05). Different lowercase letters in the same row mean statistically different values according to the Tukey’s test (P < 0.05).

**Table S2**. Effect of bile salts (0.1, 0.2, and 0.3%) on the viability (log CFU/mL) of *L. lactis* (L1 and L2) and *E. faecium* (EF).

| **Strain** | **Bile (%)** | **Time** | | | |
| --- | --- | --- | --- | --- | --- |
|  |  | 0h | 2h | 4h | 6h |
| L1 | Control | 9.48 ± 0.08 ^ABa^ | 9.37 ± 0.16 ^Aa^ | 8.00 ± 0.00 ^Cb^ | 7.54 ± 0.06 ^Dc^ |
|  | 0.1 | 9.27 ± 0.27 ^ABC^ | - | - | - |
|  | 0.2 | 6.78 ± 0.18 ^E^ | - | - | - |
|  | 0.3 | 6.00 ± 0.00 ^F^ | - | - | - |
|  |  |  |  |  |  |
| L2 | Control | 9.15 ± 0.11 ^ABCa^ | 9.09 ± 0.09 ^ABa^ | 9.10 ± 0.02 ^ABa^ | 8.19 ± 0.04 ^Cb^ |
|  | 0.1 | 8.95 ± 0.05 ^Ca^ | 8.59 ± 0.59 ^Ba^ | 9.09 ± 0.09 ^ABa^ | 9.05 ± 0.10 ^ABa^ |
|  | 0.2 | 7.14 ± 0.06 ^DEb^ | 9.02 ± 0.06 ^ABa^ | 8.99 ± 0.09 ^ABa^ | 9.08 ± 0.04 ^ABa^ |
|  | 0.3 | 7.31 ± 0.14 ^Dc^ | 9.14 ± 0.06 ^Aa^ | 8.93 ± 0.03 ^Bb^ | 9.19 ± 0.01 ^Aa^ |
|  |  |  |  |  |  |
| EF | Control | 9.65 ± 0.29 ^Aa^ | 9.08 ± 0.04 ^ABb^ | 9.02 ± 0.10 ^ABb^ | 8.98 ± 0.05 ^ABb^ |
|  | 0.1 | 9.08 ± 0.07 ^BCa^ | 9.10 ± 0.07 ^ABa^ | 9.13 ± 0.05 ^Aa^ | 8.90 ± 0.04 ^Bb^ |
|  | 0.2 | 9.00 ± 0.08 ^Ca^ | 8.95 ± 0.07 ^ABa^ | 9.00 ± 0.08 ^ABa^ | 8.83 ± 0.20 ^Ba^ |
|  | 0.3 | 9.02 ± 0.05 ^Ca^ | 8.92 ± 0.18 ^ABa^ | 8.90 ± 0.10 ^Ba^ | 9.00 ± 0.09 ^ABa^ |

The results are expressed as means ± standard deviations n = 3; (-) indicates that the counts
were < 100 CFU/mL. Different uppercase letters in the same column mean statistically different values according to the Tukey’s test (p < 0.05). Different lowercase letters in the same row mean statistically different values according to the Tukey’s test (p < 0.05).
